# miRNome Profiling Reveals Shared Features in Breast Cancer Subtypes and Highlights miRNAs That Potentially Regulate MYB and EZH2 Expression

**DOI:** 10.3389/fonc.2021.710919

**Published:** 2021-09-27

**Authors:** Stephany Corrêa, Francisco P. Lopes, Carolina Panis, Thais Basili, Renata Binato, Eliana Abdelhay

**Affiliations:** ^1^ Centro de Transplante de Medula Óssea (CEMO), Instituto Nacional de Câncer (INCA), Rio de Janeiro, Brazil; ^2^ Grupo de Biologia do Desenvolvimento e Sistemas Dinâmicos, Universidade Federal do Rio de Janeiro (UFRJ), Duque de Caxias, Brazil; ^3^ Laboratório de Biologia de Tumores, Universidade Estadual do Oeste do Paraná (UNIOESTE), Francisco Beltrão, Brazil; ^4^ Department of Pathology, Memorial Sloan Kettering Cancer Center, New York, NY, United States

**Keywords:** miRNome, breast cancer, MYB, EZH2, novel miRNAs

## Abstract

Breast cancer (BC) has been extensively studied, as it is one of the more commonly diagnosed cancer types worldwide. The study of miRNAs has increased what is known about the complexity of pathways and signaling and has identified potential biomarkers and therapeutic targets. Thus, miRNome profiling could provide important information regarding the molecular mechanisms involved in BC. On average, more than 430 miRNAs were identified as differentially expressed between BC cell lines and normal breast HMEC cells. From these, 110 miRNAs were common to BC subtypes. The miRNome enrichment analysis and interaction maps highlighted epigenetic-related pathways shared by all BC cell lines and revealed potential miRNA targets. Quantitative evaluation of BC patient samples and GETx/TCGA-BRCA datasets confirmed *MYB* and *EZH2* as potential targets from BC miRNome. Moreover, overall survival was impacted by *EZH2* expression. The expression of 15 miRNAs, selected according to aggressiveness of BC subtypes, was confirmed in TCGA-BRCA dataset. Of these miRNAs, miRNA-mRNA interaction prediction revealed 7 novel or underexplored miRNAs in BC: miR-1271-5p, miR-130a-5p, and miR-134 as *MYB* regulators and miR-138-5p, miR-455-3p, miR-487a, and miR-487b as *EZH2* regulators. Herein, we report a novel molecular miRNA signature for BC and identify potential miRNA/mRNAs involved in disease subtypes.

## Introduction

Breast cancer (BC) is the most common type of cancer and the leading cause of cancer-related death among women worldwide. As a result of several etiologic factors and clinical characteristics, BCs have different prognoses and treatments. BC is classified according to the presence of receptors into 4 major molecular subtypes (1, 2): luminal A (estrogen and/or progesterone receptor positive), luminal-HER (estrogen and/or progesterone receptor positive and HER2 positive), HER2 (human epidermal receptor 2 positive or enriched) and triple-negative (negative for these receptors) (3).

The carcinogenesis of BC is complex and involves several distinct mechanisms at the cellular and molecular levels. In addition to genetic alterations and microenvironment involvement, epigenetic modifications also occur in tumor cell progression. These alterations in gene expression can be driven by different processes, such as methylation, histone modification, chromatin remodeling, and by noncoding RNAs, such as microRNAs (miRNAs) (4, 5).

MiRNAs are a class of small noncoding RNAs approximately 22 nucleotides in length that have an important role in posttranscriptional gene regulation by targeting messenger RNAs (mRNAs) with an antisense complementary sequence, thus inhibiting or inducing the translation of the mRNA by different mechanisms (6). MiRNAs can target not just one but several mRNAs due to imperfect base pairing and are key regulators in various cellular processes, including cellular growth, differentiation and apoptosis. They are also deregulated in many diseases, including cancer (7). Because of their contributions to gene regulation and their involvement in several signaling networks, these molecules can act as tumor suppressor or oncogenic miRNAs by targeting oncogenes or tumor suppressor genes, respectively.

MiRNAs have been actively studied in recent years (8), and some studies have observed aberrant expression of miRNAs in BC. Different groups have observed the overexpression of miR-21 and miRNA-221/222, which confer trastuzumab and tamoxifen resistance, respectively (9, 10). In contrast, members of the let-7 family are downregulated (11). Most of these studies focused on one or a few miRNAs, or on a specific pathway of interest, as there are many targets, elements and interactions affected by miRNA regulation.

Genomic technologies can provide information to better understand the biology and the components involved in tumorigenesis. Although BC classification provides important information regarding the underlying biology and clinical behavior of cancer, which provides insights into its prognosis and the appropriate treatment approach (12), it is still difficult to transpose transcriptomic and proteomic studies in BC; this is likely due to the differential expression of miRNAs (13, 14).

Moreover, it has become clear that the global screening of miRNAs may offer valuable information, which could be used to enhance the analysis of already existing BC data. MiRNA expression profiling has the potential to improve tumor stratification, patient diagnosis, and the assignment of patient prognoses and even has the potential to be used as a therapeutic tool. To achieve this impact, high-throughput approaches are applied to identify cancer-specific molecular-fingerprints by screening thousands of targets (15, 16).

Therefore, in the present work, we analyzed the miRNome of cellular lineages that represent the BC subtypes to identify the signaling and pathways deregulated in BC, together with potential biomarkers of the disease.

## Materials And Methods

### Cell Culture

In this study, cellular lineages were used to model different BC subtypes. The cell lines MCF-7, EVSA-T (luminal) and MDA-MB-231 (triple-negative) were obtained commercially from DSMZ collection, with the following catalog numbers: ACC-115, ACC-433, and ACC-732, respectively. The HCC-1954 (HER2) cell line was purchased from ATCC bioresource (ATCC^®^ CRL-2338^™^). The cells were cultured in appropriate conditions using RPMI-1640 medium (Sigma-Aldrich) supplemented with 10% fetal bovine serum (FBS) (Thermo Fisher Scientific), 1% glutamine (Thermo Fisher Scientific) and 1% penicillin/streptomycin (Sigma-Aldrich). The cell line used as a control, HMEC (human mammary epithelial cells), was obtained from Thermo Fisher Scientific (catalog number A10565), and the cells were maintained in the recommended culture medium (HuMec) (Thermo Fisher Scientific) according to the manufacturer’s instructions. All of the cultures were maintained at 37°C in a 5% CO_2_ air atmosphere.

### Patient Cohort

This study was designed and conducted in accordance with the ethical principles from the Declaration of Helsinki and participants signed informed consent forms. Approval for this study was obtained by the Institutional Ethics Committee from the Brazilian National Cancer Institute – (INCA, Rio de Janeiro, Brazil) (CAAE number: 48492915.0.0000.5274). For the validation cohort, patient samples were obtained from the National Bank of Tumors and DNA (BNT) at INCA.

### miRNA Enrichment and Quantification

An enriched fraction of miRNAs from the cell lines was obtained using a miRNeasy Mini kit (Qiagen), and the quantification of miRNAs was performed with a Qubit microRNA Assay Kit (Life Technologies). Reverse transcription was performed using miScript II RT (Qiagen), according to the concentration of each sample and the manufacturer’s instructions.

### Human miRNome miScript™ miRNA PCR Array

The expression of miRNA was evaluated by quantitative real-time PCR (qRT-PCR) using a Human miRNome miScript™ miRNA PCR Array, which was performed on a Rotor-Gene using a detection system that involved a miScript SYBR Green PCR Kit and a total of 12 miScript miRNA PCR Array Rotor-Discs. All of the components were acquired from Qiagen. This technique allows for the expression analysis of the 1008 mature miRNA sequences from the human miRNA genome that are abundantly expressed and best characterized in miRBase (www.mirbase.org).

To perform the PCR array, a premix of cDNA, miScript Universal Primer, QuantiTect SYBR Green PCR Master Mix, and RNase-free water was prepared according to the manufacturer’s protocol, and then it was added to a miScript miRNA PCR Array Rotor-Disc using a QIAgility instrument. Each Rotor-Disc was tightly sealed with a Rotor-Disc Heat-Sealing Film and transferred to the Rotor-Gene Q to start the PCR cycles. The cycling program had an initial step of 15 minutes at 95°C to activate the HotStart Taq DNA polymerase, which was followed by 40 cycles of three-step cycling: denaturation at 94°C for 15 seconds, annealing at 55°C for 30 seconds, and extension at 70°C for 30 seconds. Each Rotor-Disc contained different housekeeping genes for normalization by arithmetic mean and different controls. A cycle threshold (CT) value of 0.02 was used to analyze the data according to the protocol. CT values were inserted into the miScript miRNA PCR Array Data Analysis Excel Template available at http://pcrdataanalysis.sabiosciences.com/mirna. This tool automatically performed relative quantification of the expression of genes of interest in MCF-7, EVSA-T, HCC-1954 and MDA-MB-231 cell lines and compared their levels with those of HMEC cells (control lineage), using the ΔΔCT method (17).

### MetaCore™ and Venn Diagram Analysis

Lists containing differentially expressed (DE) miRNAs with a fold change ≥ 2 derived from each lineage were uploaded into MetaCore™ (GeneGO Inc.) for analysis. Different analyses were performed, including a pooled analysis with data from all four cell lines. The online software allowed for functional analysis and categorization of the differentially expressed miRNAs into signaling pathways and networks (occasionally MetaCore™ do not distinguish -5p and -3p isoforms), and it yields information regarding potential miRNA targets. The Venn diagram was drafted with an online tool: http://bioinformatics.psb.ugent.be/webtools/Venn/.

### Real Time Quantitative PCR

Total RNA was extracted from BC cell lines, tissues from BC patients and healthy control samples using TRIzol reagent (Invitrogen) and a RNeasy mini kit (Qiagen). Total RNA was treated with a DNase Amplification Grade I Kit (Invitrogen) to remove DNA contamination. Complementary DNA synthesis was performed with Superscript-II Reverse Transcriptase (Invitrogen) following the manufacturer’s protocol. RT-qPCR was performed using SYBR Green Master Mix (Invitrogen) in a Rotor-Gene Q (Qiagen). The following forward (Fow) and reverse (Rev) primers were used: *MYB* - Fow 5’ AGTCTGGAAAGCGTCACTTG 3’, Rev 5’ GTTCCATTCTGTTCCACCAG 3’; *EZH2* - Fow 5’ AGAAGGGACCAGTTTGTTGG 3’, Rev 5’ GTGCACAGGCTGTATCCTTC 3’; *MITF* - Fow 5’ TTGGGCTTGATGGATCCTGC 3’, Rev 5’ GGCAGACCTTGGTTTCCAT 3’; *SIP1* - Fow 5’ CCCTTCTGCGACATAAATACGA 3’, Rev 5’ TGTGATTCATGTGCTGCGAGT 3’; and *GAPDH* - Fow 5’ GTCAACGGATTTGGTCGTATTG -3′, Rev 5’ TGGAAGATGGTGATGGGATTT - 3’. The PCR cycling conditions included an initial denaturation of 95°C for 10 minutes, followed by 45 cycles of 20 seconds at 95°C, 20 seconds at 60°C, and 40 seconds at 72°C. The GAPDH mRNA levels were used as a reference of expression. The fold-expression was calculated according to the ΔΔCT method (17).

### mRNA and miRNA Expression Data From The Cancer Genome Atlas

A total of 1046 cases were extracted from The Cancer Genome Atlas (TCGA) breast cancer repository at the Genomic Data Commons (GDC) Data Portal (https://portal.gdc.cancer.gov) for mRNA expression analysis. The following parameter selections were adopted: Program TCGA; Project TCGA-BRCA; Disease Type Ductal and Lobular Neoplasms; Sample Type - primary tumor or solid tissue normal; Gender Female; Data Category Transcriptome Profiling; Data type Gene Expression Quantification; Experimental Strategy RNA-Seq; and Workflow Type HTSeq – FPKM.

The same strategy was used to obtain a total of 1037 cases from TCGA-BRCA for miRNA expression analysis. The following parameter selections were adopted: Program TCGA; Project TCGA-BRCA; Disease Type Ductal and Lobular Neoplasms; Sample Type - primary tumor or solid tissue normal; Gender Female; Data Category Transcriptome Profiling; Data type Isoform Expression Quantification; Experimental Strategy miRNA-Seq; and Workflow Type BCGSC miRNA Profiling (RPM).

The TCGA-BC dataset was categorized according to the BC molecular subtypes by the cBioPortal online tool (https://www.cbioportal.org/) and TCGABiolinks (R/Bioconductor package) (18). Patients with DCIS (ductal in situ), neoadjuvant therapy, prior treatment/malignancy, inconclusive classification and Normal-like subtype data, were further excluded from all our analyses, together with their paired normal solid tissue.

### mRNA Expression Data From Genotype-Tissue Expression

Healthy breast tissue samples (n=179) from the public resource Genotype-Tissue Expression (GTEx) project were sampled using the UCSC Xena online tool (https://xena.ucsc.edu). mRNA expression data was obtained by RNAseq strategy (FPKM) and compared to TCGA-BRCA data with TCGABiolinsksal (R/Bioconductor package) (19).

### miRNA-mRNA Target Prediction

The prediction of miRNA-mRNA interaction binding sites was performed with the following online tools: miRTAR (http://mirtar.mbc.nctu.edu.tw/human/), miRwalk (http://mirwalk.umm.uni-heidelberg.de), Targetscan (http://www.targetscan.org/vert_72/), miRnet (https://www.mirnet.ca), miRDB (http://mirdb.org), and miRror (http://www.proto.cs.huji.ac.il/mirror/search.php). Using these tools, the 5’ untranslated region (5’UTR), coding sequence (CDS) and 3’ untranslated region (3’UTR) were investigated from selected mRNAs of interest.

### Statistical Analysis

RT-qPCR experiments were performed in triplicate, and the expression data were analyzed with GraphPad Prism^®^ v.8 software (GraphPad) and presented as the mean ± SD. Patient expression data were categorized in quartiles (low - 25% downregulated; high - 25% overexpressed) and Kaplan-Meyer analysis was used to access death risk (overall survival). Data for patients who did not present complete information regarding days-to-death/days-to-follow-up or were followed fewer 30 days were removed from the study. Statistical analyses (ANOVA for expression data and Gehan-Breslow-Wilcoxon test for overall survival) were performed using GraphPad Prism^®^ v.8 software (GraphPad). P-values (p) <0.05 were considered statistically significant (*p < 0.05, **p < 0.01, ***p < 0.001, and ****p < 0.0001).

## RESULTS

### *In Vitro* Breast Cancer Models Exhibit a Shared miRNA Profile Based on miRNome

**Figure 1:**
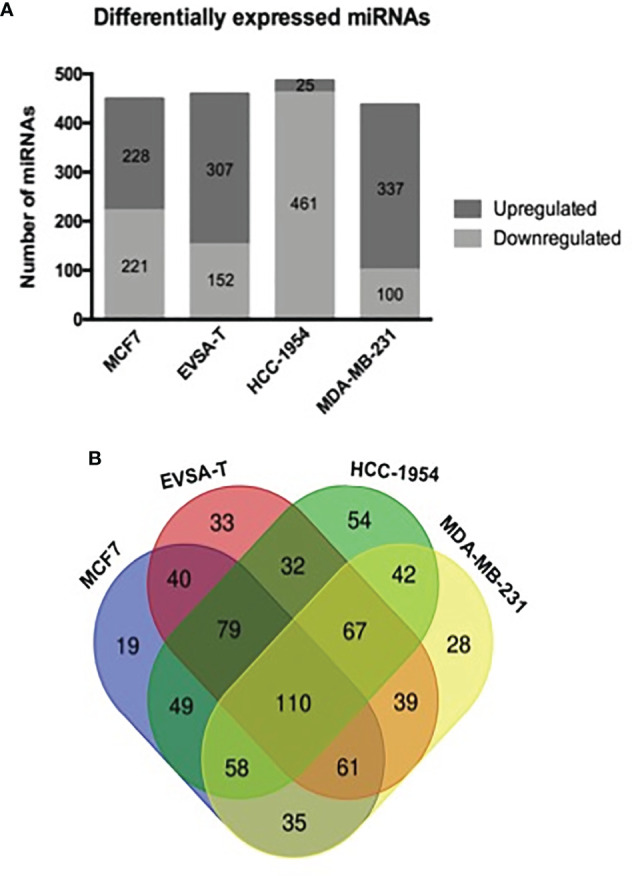
Differentially expressed miRNAs in human breast cancer cell lines. **(A)** Number of miRNAs obtained as differentially expressed by RT-qPCR analysis of 1008 miRNA levels from miRNome PCR array. SnoRNAs/snRNAs were used as housekeeping genes for normalization. Positive and negative controls were also evaluated in the array. Quantification was performed by the ΔΔCT method. The results were normalized relative to the HMEC cell line. **(B)** A Venn diagram drafted from http://bioinformatics.psb.ugent.be/webtools/Venn, showing the total number and overlapping miRNAs identified as differentially expressed by quantitative PCR array from the following breast cancer cell lines: MCF7, EVSA-T, HCC-1954 and MDA-MB-231 (normalized relative to the HMEC cell line).

To obtain the miRNome profiles from the BC cell lines used in our study (MCF7, EVSA-T, HCC-1954 and MDA-MB-231), we performed a global analysis of the 1008 miRNAs in the miRNome PCR array. According to our results, the following number of miRNAs was identified as DE: 449 miRNAs in MCF7 (221 downregulated, 228 upregulated), 459 in EVSA-T (152 downregulated, 307 upregulated), 486 in HCC-1954 (461 downregulated, 25 upregulated) and 437 in MDA-MB-231 (100 downregulated, 337 upregulated). The miRNAs levels of all BC cell lines were compared to the normal cell line HMEC, which was used as a control, with a ≥ 2-fold-change as a cut-off (**Figure 1A**). The DE miRNAs from each cell line with their respective fold-change are listed in **Tables S1**–**S4**. To highlight the potential miRNAs involved in BC carcinogenesis, a Venn diagram was drafted. From this diagram, we identified 110 common differentially expressed miRNAs when comparing the BC cell lines analyzed in our study with the HMEC cell line. The diagram also identified miRNAs exclusive to each subtype (**Figure 1B**).

As our goal was to understand the signaling and pathways related to BC and to correlate miRNA expression with BC subtypes, we performed an *in silico* analysis using these 110 common DE miRNAs. Cell lines were designated with numbers (1 to 4) according to their subtypes (MCF7, EVSA-T, HCC-1954 and MDA-MB-231) to facilitate the analysis. MetaCore™ provided an overview regarding the 10 most relevant pathway maps from our data (**Table 1**). Some of the pathways are well-known in BC, such as TGFβ signaling and epithelial-to-mesenchymal transition (EMT), as well as pathways involved in altering cell proliferation, survival, migration and angiogenesis. Moreover, epigenetic alterations mediated by miRNAs also appeared among the top 10 altered pathways.

**Table 1:** Top 10 pathway maps from 110 common miRNAs in the BC miRNome.

110 miR	Pathways
1	Role of epigenetic alterations in proliferation and differentiation of SCLC cells
2	Role of microRNA in cell proliferation on colorectal cancer
3	TGF-beta signaling *via* microRNA in breast cancer
4	Regulation of microRNAs in colorectal cancer
5	microRNA-dependent inhibition of EMT
6	Role of microRNA in cell migration, survival and angiogenesis in colorectal cancer
7	Role of epigenetic alterations in survival and migration of SCLC cells
8	microRNA in prostate cancer
9	Hypothetical role of microRNAs in fibrosis development after myocardial infarction
10	MicroRNAs in melanoma

Particular miRNAs appeared in more than one pathway map; e.g., miRNA-200b, miRNA-497-5p and miRNA-34b-3p, confirming that they are important and the most studied miRNAs. The miRNAs that may be related to proliferation, cell survival and EMT pathways were differentially expressed in the *in vitro* models. As shown, we observed that miR-497-5p and miR-95 (**Figure S1**) were upregulated in BC, while miR-205-5p was downregulated (**Figure S2**). As shown in **Figure S2**, we observed a shift in miRNA expression; e.g., miR-200a, miR-200c, miR-141 and miR-429. These were upregulated in less aggressive cell lines and downregulated in more aggressive cell lines, implying the differential regulation of the *SIP1* gene.

Interaction maps (networks) generated by MetaCore™ were also based on described and validated interactions, allowing identification of known relevant targets in BC, such as PTEN (**Figure S3A**). Nevertheless, the interaction map of miR-205-5p (**Figure S3B**) showed that this miRNA might be directly epigenetically regulated by the lncRNA LINC-ROR and indirectly regulated by EZH2, which has an important role in chromatin remodeling.

### *MYB* and *EZH2* Are Potential Targets Identified in the Breast Cancer miRNome

**Figure 2:**
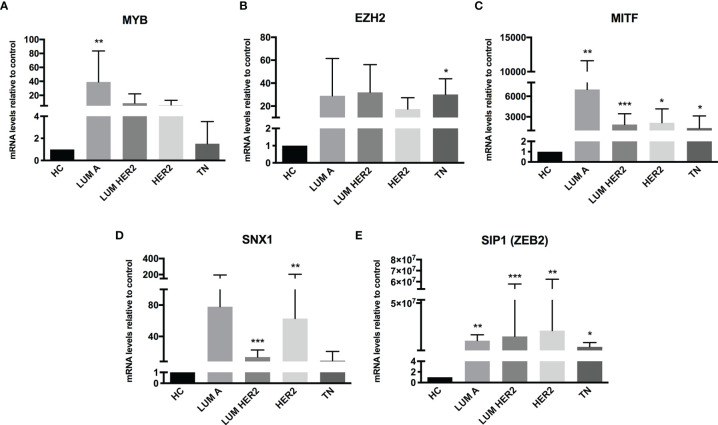
Putative miRNA targets are differentially expressed in BC patients. RT-qPCR analysis of miRNA targets in BC patients. The mRNA level of **(A)** *MYB*, **(B)** *EZH2*, **(C)** *MITF*, **(D)** *SNX1* and **(E)** *SIP1 (ZEB2)* was assessed by raw expression values normalized to GAPDH expression. Healthy donors were used as healthy controls (HC n = 5). Quantification was performed by the ΔΔCT method. Statistical differences are related to the HC group (LUM A n = 5; LUM HER2 n = 12; HER2 n = 7; TN n = 4). HC, Healthy Control; TN – Basal. *p < 0.05, **p < 0.01 and ***p < 0.001.

**Figure 3:**
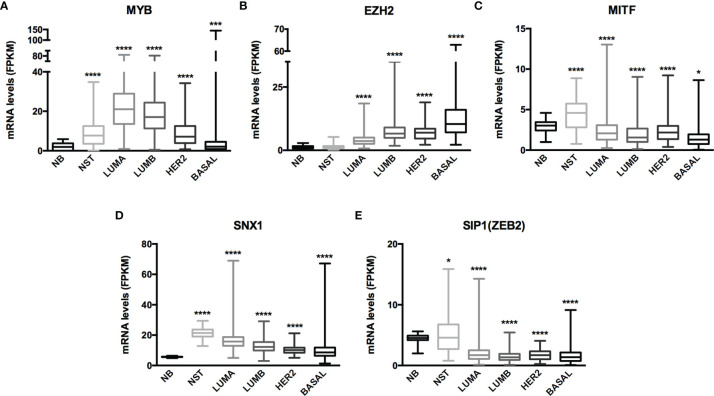
mRNA expression from GTEx and TCGA-BRCA dataset evidenced MYB and EZH2 as miRNome targets. Histogram analysis of miRNAs targets from the GTEx/TCGA-BRCA dataset. We collected **(A)** *MYB*, **(B)** *EZH2*, **(C)** *MITF*, **(D)** *SNX1* and **(E)** *SIP1(ZEB2)* mRNA levels (FKPM) from NB, NST, LUMA, LUMB, HER2 and BASAL subtypes. Statistical differences are related to the NB group. NB, Normal Breast; NST, Normal Solid Tissues. *p < 0.05 and ****p < 0.0001.

**Figure 4:**
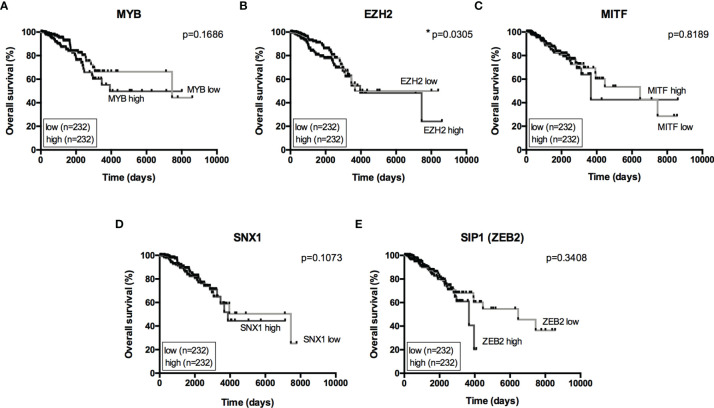
Overall Survival analysis from miRNome targets. Kaplan-Meier curves for overall survival in the TCGA-BRCA dataset with low- and high-expression of **(A)** *MYB*, **(B)** *EZH2*, **(C)** *MITF*, **(D)** *SNX1* and **(E)** *SIP1(ZEB2)*. Expression levels from LUMA, LUMB, HER2 and BASAL subtypes were determined as low or high based on quartile analysis. Statistical differences show poor overall survival (higher risk) for patients with a specific mRNA expression. *p < 0.05.

The miRNA targets first identified by MetaCore™ were targets commonly described in cancer studies, such as p53 and c-myc, among others. Moreover, the pathways addressed genes that are regulated and that may regulate miRNA expression. Nevertheless, when we examined these pathways more closely, we were able to address some poorly investigated (direct and indirect) targets in BC, such as SNX1. The following targets were selected based on miRNA pathway maps and networks from MetaCore™ analysis, together with chip array data (BC patients) from our previous work (data not shown) (20): *MYB, EZH2, MITF, SNX1*, and *SIP1.* Although the latter is recognized as relevant for EMT, and for this matter is relevant in BC, the ultimate question here is whether this gene may be regulated by miRNAs other the well-described ones. We performed a quantitative analysis in an independent cohort of healthy control donors (n=5) and BC molecular subtypes (total n=28). As shown in **Figure 2**, most of the gene profiles were upregulated in BC subtypes, suggesting that they are relevant in BC and are potential miRNA targets. Moreover, the expression of these genes was investigated using the TCGA dataset. The TCGA-BRCA consortium accounts for 1098 cases from which 1046 were extracted according to the applied filters. A total of 106 normal solid tissues (NST *- adjacent tissue*) were obtained and 975 primary tumors were categorized by BC molecular subtypes, as follows: 527 from LUMA, 193 from LUMB, 80 from HER2 and 175 from BASAL (TN). Healthy breast (normal breast - NB) samples from the GTEX dataset were also evaluated (n=179). As shown in **Figure 3**, there is a correspondence in expression levels for *MYB* and *EZH2* among BC subtypes when compared to NST and NB. Moreover, using the same dataset, overall survival analysis from quartiles showed that *MYB* and *EZH2* overexpression are related to survival rate (**Figure 4**), with the latter statistically significant, which supports their relationship in BC subtypes.

### A Set of 7 miRNAs May Be Related to *MYB* and *EZH2* Expression in Breast Cancer

**Figure 5:**
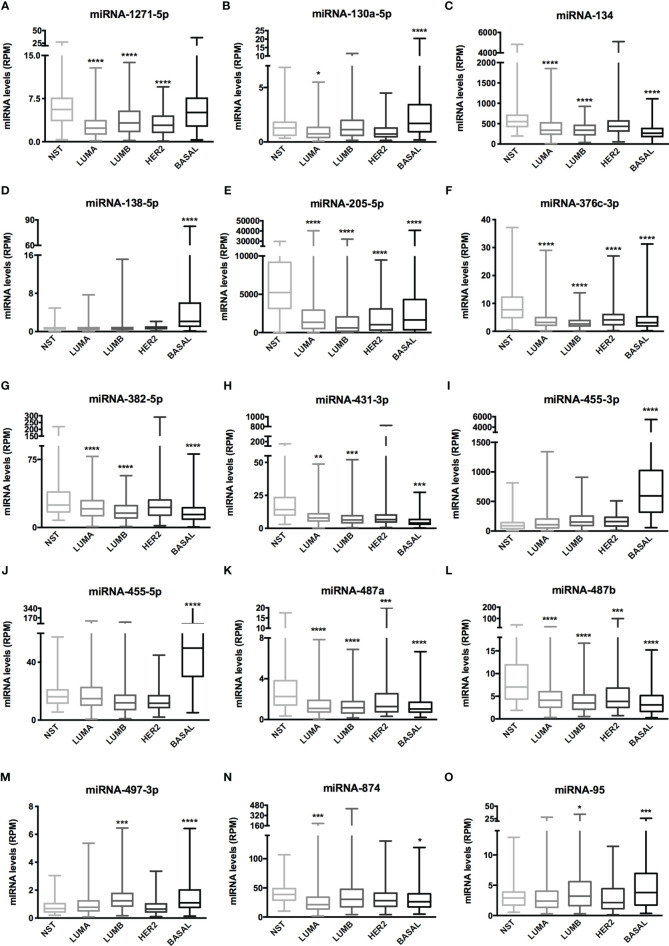
miRNA expression of 15 miRNAs related to BC aggressiveness from TCGA dataset. Histogram analysis of miRNAs targets from the TCGA-BRCA dataset. **(A)** miRNA-1271-5p, **(B)** miRNA-130a-5p, **(C)** miRNA-134, **(D)** miRNA-138-5p, **(E)** miRNA-205-5p, **(F)** miRNA-376c-3p, **(G)** miRNA-382-5p, **(H)** miRNA-431-3p, **(I)** miRNA-455-3p, **(J)** miRNA-455-5p, **(K)** miRNA-487a, **(L)** miRNA-487b, **(M)** miRNA-497-3p, **(N)** miRNA-874, **(O)** miRNA-95. We collected miRNA expression data (RPM) of interest from NST, LUMA, LUMB, HER2 and BASAL subtypes. Statistical differences are related to NST group. NST, Normal Solid Tissues. *p < 0.05, **p < 0.01, ***p < 0.001, and ****p < 0.0001.

**Figure 6:**
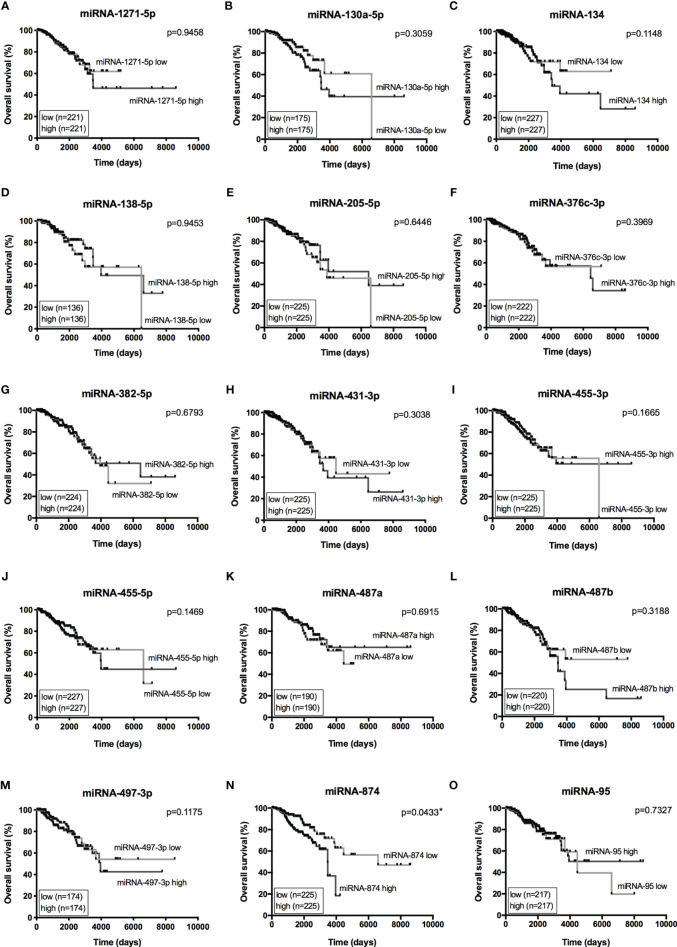
Overall Survival curves from 15 miRNAs of interest. Kaplan-Meier curves for overall survival of TCGA-BRCA dataset with low- and high-expression of 15 miRNAs of interest. **(A)** miRNA-1271-5p, **(B)** miRNA-130a-5p, **(C)** miRNA-134, **(D)** miRNA-138-5p, **(E)** miRNA-205-5p, **(F)** miRNA-376c-3p, **(G)** miRNA-382-5p, **(H)** miRNA-431-3p, **(I)** miRNA-455-3p, **(J)** miRNA-455-5p, **(K)** miRNA-487a, **(L)** miRNA-487b, **(M)** miRNA-497-3p, **(N)** miRNA-874, **(O)** miRNA-95. Expression levels from LUMA, LUMB, HER2 and BASAL subtypes were determined as low or high based on quartile analysis. Statistical differences show poor overall survival (higher risk) for patients with a specific miRNA expression (high or low). *p < 0.05.

The Venn diagram pointed 110 DE miRNAs as common to all molecular subtypes and for this reason as potentially directly involved in BC tumorigenesis. However, their fold change varied among cell lines. This aspect is extremely relevant because, even though we analyzed the same miRNAs for pathway and interactome network analysis, fold change differences highlight a potential signature regarding BC subtypes. Therefore, we filtered the 110 DE miRNA list and selected those miRNAs upregulated and downregulated in all the cell lines and miRNAs whose expression shifted according to subtypes. As shown in **Table S5**, 8 miRNAs were upregulated in all BC models; 19 were downregulated in all BC models and 27 miRNAs presented initial (e.g., MCF7 cell line) up- or downregulation then later exhibited down- or upregulation (e.g., MDA-MB-231 cell line). The expression of these 54 miRNAs was also investigated in the TCGA dataset using a total of 97 normal solid tissues and 960 primary tumor samples. These primary tumors were also categorized by BC molecular subtypes as follow: 524 from LUMA, 189 from LUMB, 79 from HER2 and 168 from BASAL (TN). As GTEX data does not separate -5p and -3p isoforms, we did not analyze NB samples for miRNAs. As shown in **Figure 5**, the expression levels of 15 miRNAs corroborated with our *in vitro* models for all BC subtypes, regardless of upregulation, downregulation or shift in expression (**Figure S4**). Although only miRNA-874 exhibited significant differences in overall survival rates from low/high-expressed quartiles (**Figure 6**), most Kaplan-Meyer curves correlated with gene expression e.g., miR-205-5p, miR-382-5p, miR-487a (low expression - worse survival rate) and miR-497-3p (high expression - worse survival rate). These 15 miRNAs were also analyzed *via in silico* prediction of the potential targets evidenced by BC patient quantitative analysis - *MYB*, and *EZH2.* For this analysis, 6 online tools were used for miRNA-mRNA interaction for the investigation of validated and predicted sites. Our results showed that most predicted sites are localized in the CDS; however, sites in the 5UTR and 3UTR were also identified. At least one predicted binding site was found for 14 miRNAs; only miR-376c-3p did not present any consensus-binding site in both *MYB* and *EZH2* transcripts (NM_001130173 and NM_004456, respectively) (**Table 2**). Most miRNA binding sites were predicted for mRNA sequences but validated sites were also observed in our analysis. For *MYB*, 4 miRNAs presented 3UTR binding sites identified by at least 2 types of software (miR-1271-5p, miR-130a-5p, miR-134 and miR-497-3p). The latter is upregulated in our study, so it does not correspond with *MYB* expression. For *EZH2*, miR-138-5p had binding sites identified in 5 software packages, and most miRNAs with 3UTR binding sites were downregulated in BC subtypes (miR-455-3p, miR-487a and miR-487b). The exception is miR-95; further, miR-497-3p levels does not correspond to *EZH2* expression.

**Table 2:** Prediction analysis of miRNA-target interaction from MYB and EZH2 mRNAs.

	miRTAR	miRwalk	Targetscan	miRnet	miRDB	miRor
**CMYB**						
**hsa-miR-1271-5p**		CDS	3UTR		x	
**hsa-miR-130a-5p**		3UTR				x
**hsa-miR-134**	3UTR	3UTR, CDS				
**hsa-miR-138-5p**						x
**hsa-miR-205-5p**		CDS				
**hsa-miR-376c-3p**						
**hsa-miR-382-5p**		CDS				
**hsa-miR-431-3p**		3UTR, CDS				
**hsa-miR-455-3p**		3UTR, CDS				
**hsa-miR-455-5p**						
**hsa-miR-487a**		CDS				
**hsa-miR-487b**						
**hsa-miR-497-3p**		CDS	3UTR		x	x
**hsa-miR-874**	CDS	CDS				
**hsa-miR-95**		CDS	3UTR			
**EZH2**						
**hsa-miR-1271-5p**		CDS				
**hsa-miR-130a-5p**		CDS				
**hsa-miR-134**		5UTR				
**hsa-miR-138-5p**		CDS	3UTR	x	x	x
**hsa-miR-205-5p**		CDS				
**hsa-miR-376c-3p**						
**hsa-miR-382-5p**						
**hsa-miR-431-3p**		CDS				
**hsa-miR-455-3p**	3UTR	CDS				
**hsa-miR-455-5p**		CDS				
**hsa-miR-487a**	3UTR	CDS				
**hsa-miR-487b**	3UTR	CDS				
**hsa-miR-497-3p**		CDS				
**hsa-miR-874**	CDS	5UTR				
**hsa-miR-95**			3UTR		x	

(x) - online tool where the miRNA-mRNA interaction was found.

Therefore, based on our results, we can suggest a set of 7 miRNAs investigated in BC that may act in balance to promote tumorigenesis through targeting *MYB* (miR-1271-5p, miR-130a-5p and miR-134) and *EZH2* (miR-138-5p, miR-455-3p, miR-487a and miR-487b).

## Discussion

The attempts to better understand the mechanisms involved in cancer initiation, progression and resistance are never-ending. The knowledge acquired so far has allowed little translation from basic research to clinical medicine; however, the information has provided improvement in patient prognosis, which leads to and motivates further understanding of cancer mechanisms, until the long sought-after goal of personalized medicine is achieved (18).

In this context, the pursuit of molecules capable of adding information to current data that are applicable as diagnostic, prognostic, disease monitoring and resistance markers is what drives large-scale studies on cancer today (21, 22). Therefore, miRNAs are highly attractive because of their role in transcriptional and translational regulation. MiRNA research implies a high level of complexity regarding the molecular biology of miRNAs, as one target can be regulated by several miRNAs and the same miRNA may regulate several targets. Because of this complexity, the major reports in the literature focus on one specific miRNA or a family of miRNAs in a given query. As a result, the global study of miRNAs or the miRNome need to be the focus of more study, especially as the understanding of global modifications in cell biology may lead to or be involved in cancer. Therefore, the aim of this study was to perform miRNome profiling of BC using *in vitro* models that correspond to the four major molecular subtypes to identify the signaling and pathways related to BC. In parallel, our goal was to reveal novel miRNAs (and targets) that could be associated with BC tumorigenesis.

With a PCR array approach, we identified more than 400 DE miRNAs among BC subtypes. As the amount of data was massive, we executed *in silico* analysis with Mecatore™ software to categorize the 110 common differentially expressed miRNAs among the cell lines to better understand their role in BC tumorigenesis. In a general analysis where DE miRNAs from each cell line were uploaded and evaluated alone, the top 10 pathway maps identified by MetaCore™ varied little among BC subtypes (data not shown). The same pathways were obtained from enrichment analysis of the 110 miRNAs, demonstrating that they were in fact associated with the identified signaling molecules and pathways.

Most of the miRNA pathway maps were previously described in colorectal and melanoma tumors, meaning that the same proposed regulation could be occurring in a BC context, but it should be validated to confirm such regulation. This finding highlights the lack of omics studies on miRNAs in BC compared to studies of other tumor types. Moreover, well-described miRNAs in cancer were identified in our study, ratifying their importance in tumorigenesis. To reveal more about the proposed regulation by MetaCore™, specific targets were selected based on previous chip array analysis (data not shown). Quantitative analysis from our targets in BC patients together with TCGA-BRCA evaluation (mRNA expression and overall survival) identified *MYB* and *EZH2* as relevant for the investigation of miRNA-mRNA binding sites. Higher levels of *EZH2* are correlated with a higher risk, which makes sense because upregulation of *EZH2* was more pronounced in the basal subtype. For *MYB*, higher levels are seen in luminal subtypes; therefore, lower levels correspond more with higher risk (although not statistically). Although *SNX1* was upregulated among BC subtypes when compared to NB, as the NST cohort also exhibited upregulation of *SNX1* mRNA levels, this gene was excluded from further analysis.

Comparison of common DE miRNAs suggested that the difference in fold change, determined through *in vitro* models, might correspond to disease aggressiveness, an important feature in BC. *In silico* comparison of DE miRNAs enabled a better visualization of this difference, and fold change analysis directed our attention to 54 particular miRNAs. Again, analysis from TCGA-BRCA showed that most miRNA expression matched some particular (s) subtype (s), and they must have a biological implication for them. However, as our focus was to identify differentially expressed miRNAs among BC subtypes, we filtered 15 miRNAs whose quantitative expression corresponded with the identified ones in our *in vitro* models. This choice will also be beneficial for further studies, where models for functional analysis investigation will be needed. Overall survival was also investigated for these 15 miRNAs. The best approach to understand the impact on overall survival would be to perform the same analysis separating each BC subtype. However, the small amount of some subtypes (e.g., HER2), and the lack of some miRNA expression data could negatively impact with the results.

With a clear subset of miRNAs, miRNAs-mRNAs interactions were used to demonstrate potential regulation of selected genes, and together with all of the applied analyses (MetaCore™ analysis, miRNAs-mRNAs binding sites prediction analysis, miRNA expression and targets expression), miR-1271-5p, miR-130a-5p and miR-134 were highlighted as regulators of *MYB*. miR-138-5p, miR-455-3p, miR-487a and miR-487b were, identified as regulators of *EZH2* based on our results.

In colorectal cancer, miR-1271-5p was shown to act as an OncomIR (23) by downregulating *NOXA*, resulting in a cell proliferation decrease and promotion of apoptosis. Nevertheless, its expression corroborates with our findings in most investigated tumors and it works as a tumor suppressor through different mechanisms and targets in hepatocellular cancer (24, 25), multiple myeloma (26, 27), ovarian cancer (28, 29) and pancreatic cancer (30). Its role in BC has not yet been addressed. A different scenario presents for miR-130a-5p. This miRNA has been recently reported in BC. Its downregulation was reported in patient plasma, and it was correlated with, among others, hormone receptors (31), corroborating our findings as lower expression of this miRNA was observed in luminal tumors. Moreover, its downregulation has been implicated by circVAPA sponging (32) in patient samples and BC cells. The same findings were obtained for other tumor types (33–35), where lncRNAs were investigated as competing endogenous RNA and therefore sponge miR-130a-5p with consequent downregulation. However, its relationship with *MYB* in BC still needs clarification. Of the potential regulators for *MYB*, miR-134 is the most studied. Its role as a tumor suppressor has been investigated in several tumors (36). In BC, it has been reported to play a role in drug response, cell proliferation, migration, and invasion (37, 38) through several targeted pathways, such as *STAT5B*, *KRAS*, *BDNF/TrkB*; nevertheless, *MYB* has not been explored as a potential target of miR-134.

As for *EZH2* potential regulators, miR-138-5p was the most consistent in miRNA-mRNA prediction from all analyzed miRNAs. Indeed, this regulation has been reported recently in different contexts (39, 40), including ovarian cancer (41) and prostate cancer (42). For BC, this miRNA was also found to be downregulated by Zhao and coworkers, corroborating our findings; it inhibits cell migration/invasion by targeting RHBDD1 (43). Nevertheless, miR-455-3p has not been reported as a regulator of *EZH2*. There are only 3 reports for BC with distinct roles for this miRNA. Li and coworkers reported overexpression in TNBC cell lines (44); Guo (45) and Zeng (46) reported that miR-455-3p was downregulated in BC patients, corroborating with our findings. In other cancer types, this miRNA has also been described as a tumor suppressor, supporting our quantitative findings. There are 3 studies on miR-487a in BC. Again, one reports that this molecule is an OncomIR (47), and the other 2 studies described this miRNA as a tumor suppressor (48, 49), which is consistent with what was presented in our work. This highlights the need for further studies in BC, as this miRNA has also been reported to be a tumor suppressor in other solid tumors. There is one study on miR-487b in BC, and it reports the regulation of *EZH2 via* miR-487b by overexpressing the 3UTR region of *EZH2* in BC cell lines to identify its target mRNAs (50). This finding is consistent with our data and justifies further studies to evaluate this regulation in disease.

As shown, most of these miRNAs were not reported/described in BC. They may play a role in cancer hallmarks related to BC together with well-studied ones, such as miR-200, miR-34, and miR-429 (among others), which were also identified in our study. The difficulty and complexity of studying miRNAs highlight their potential relevance and the essential need for further validation. A subset of nonreported miRNAs in BC from our data (1/3 from 110 common miRNAs) indicates the amount of effort necessary to elucidate the role of miRNA in BC. It is important to address that the proposed regulation presented in this study may be a combination of regulation by multiple miRNAs. Nevertheless, the screening of potential targets highlights the most important miRNAs as indicated by their differences in fold change among BC cell lines, which is confirmed in a larger cohort of patient samples.

The approach applied in this study presented signaling molecules and pathways involved in BC and revealed novel interactions and potential regulation regarding miRNAs in BC. Moreover, the identification of *in silico* targets, such as *MYB* and *EZH2*, suggests that less studied miRNAs could be potential players in BC subtypes.

## Data Availability Statement

The original contributions presented in the study are included in the article/**Supplementary Material**. Further inquiries can be directed to the corresponding author.

## Ethics Statement

The studies involving human participants were reviewed and approved by Institutional Ethics Committee from the Brazilian National Cancer Institute - INCA. Committee number: CAAE: 48492915.0.0000.5274. The patients/participants provided their written informed consent to participate in this study.

## Author Contributions

SC performed miRNA enrichment, validation experiments, *in silico* and statistical analysis, drafted the manuscript, participated in the study design and contributed intellectual content. FL extracted and processed the miRNAs and mRNAs expression data from TCGA dataset and revised the manuscript final version. CP supported the patients’ selection, and contributed intellectual content. TB performed cell culture and miRNome experiments. RB supported the in silico analysis and contributed intellectual content. EA conceived of the study and its design and coordination, provided the financial support, helped to draft the manuscript and critically revised the manuscript for intellectual content. All authors contributed to the article and approved the submitted version.

## Funding

This work was supported by FAPERJ (E-26/110.122/2013; E-26/110.664/2012). The obtained funding allowed the purchase of consumables and reagents necessary to carry out the experiments related to this study.

## Conflict of Interest

The authors declare that the research was conducted in the absence of any commercial or financial relationships that could be construed as a potential conflict of interest.

## Publisher’s Note

All claims expressed in this article are solely those of the authors and do not necessarily represent those of their affiliated organizations, or those of the publisher, the editors and the reviewers. Any product that may be evaluated in this article, or claim that may be made by its manufacturer, is not guaranteed or endorsed by the publisher.

## Supplementary Material

Supplementary Figure 1 Pathway map – Role of microRNAs in cell proliferation in colorectal cancer. Pathway map generated by MetaCore™ software. The lists of DE miRNAs were uploaded into the software and compared to correlate the fold change obtained in the PCR array with disease aggressiveness. The cell lines – MCF7, EVSA-T, HCC-1954 and MDA-MB-231 – were designated with numbers 1 to 4, respectively. Up-regulated miRNAs are marked with a red thermometer and downregulated with a blue thermometer. Red arrows indicate an inhibition effect, and green arrows indicate an activation effect.

Supplementary Figure 2 Pathway map - MicroRNA-dependent inhibition of EMT. Pathway map generated by MetaCore™ software. The lists of DE miRNAs were uploaded into the software and compared to correlate the fold change obtained in the PCR array with disease aggressiveness. The cell lines - MCF7, EVSA-T, HCC-1954 and MDA-MB-231 - were designated with numbers 1 to 4, respectively. Up-regulated miRNAs are marked with a red thermometer and downregulated with a blue thermometer. Red arrows indicate an inhibition effect, and green arrows indicate an activation effect.

Supplementary Figure 3 Interaction maps in breast cancer miRNome. Representative interaction maps generated by MetaCore™ software in **(A)** PTEN network from DE miRNAs and **(B)** the interaction map of miR-205-5p. Up-egulated miRNAs are marked with red circles, and downregulated miRNAs are marked with blue circles. The ‘checkerboard’ color indicates mixed expression for the gene between files or between multiple tags for the same miRNA.

Supplementary Figure 4 Heatmap of 15 differentially expressed miRNAs among BC cell lines confirmed in BC subtypes (TCGA).

Supplementary Table 1 miRNAs differentially expressed in the MCF7 cell line.

Supplementary Table 2 miRNAs differentially expressed in the EVSA-T cell line.

Supplementary Table 3 miRNAs differentially expressed in the HCC-1954 cell line.

Supplementary Table 4 miRNAs differentially expressed in the MDA-MB-231 cell line.

Supplementary Table 5 54 DE miRNAs according to aggressiveness among BC subtypes.

## Abbreviations

BC, breast cancer; CDS, coding sequence; DE, differentially expressed; EMT, epithelial-to-mesenchymal transition; Fow, forward; FPKM, Fragments Per Kilobase Million; GTEx, genotype-Tissue Expression; NB, normal breast; NST, normal solid tumor; Rev, reverse; RPM, reads per million; RT-qPCR, real-time quantitative PCR; TCGA, The Cancer Genome Atlas; UTR, untranslated region.
