# Supplementary figures and images for: miRNome Profiling Reveals Shared Features in Breast Cancer Subtypes and Highlights miRNAs That Potentially Regulate MYB and EZH2 Expression
Source: Front Oncol. 2021 Sep 27;11:710919. doi: 10.3389/fonc.2021.710919 (PMC8502886; doi:10.3389/fonc.2021.710919)

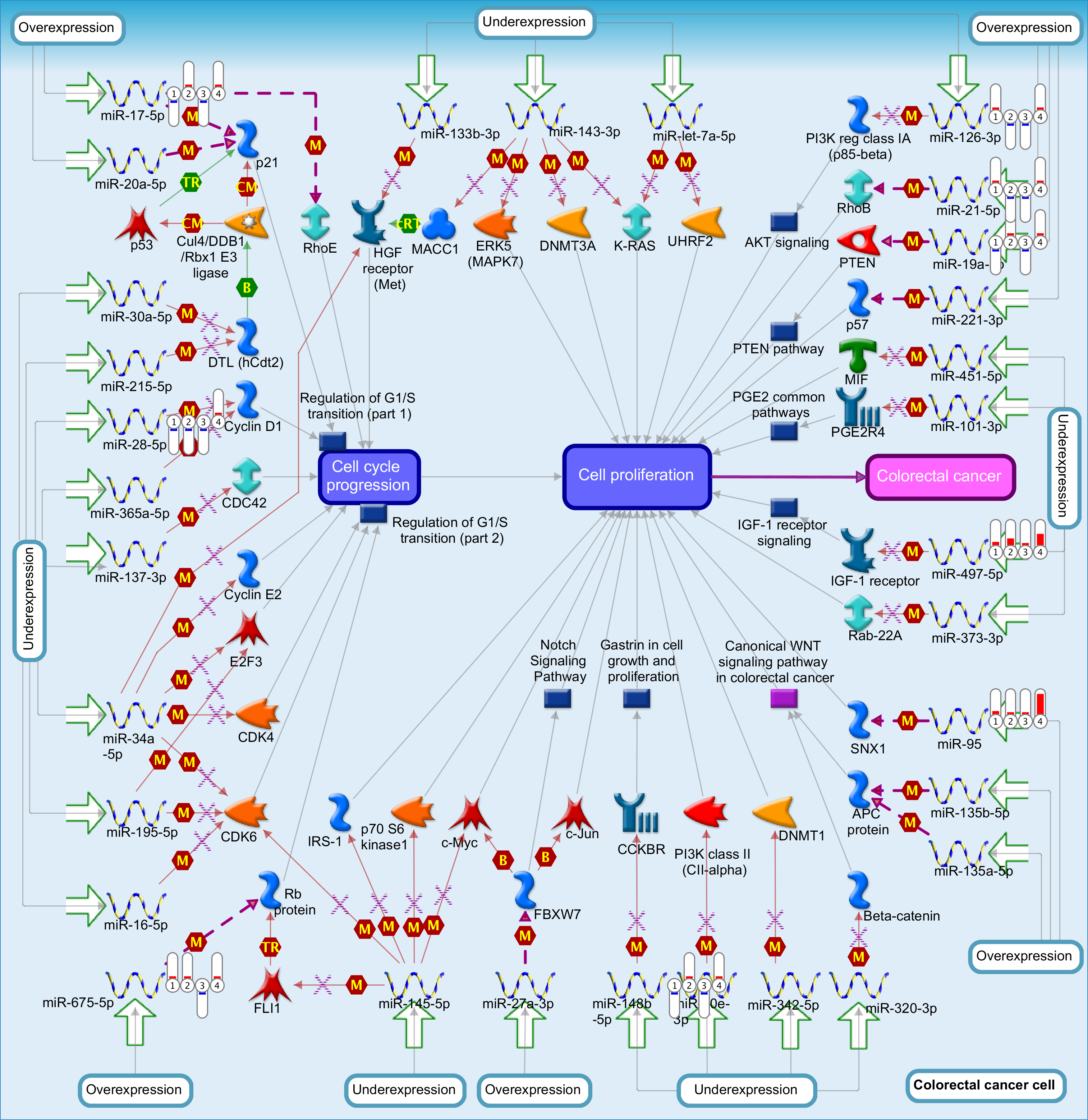

Supplement: Supplementary Figure 1 — Pathway map – Role of microRNAs in cell proliferation in colorectal cancer. Pathway map generated by MetaCore™ software. The lists of DE miRNAs were uploaded into the software and compared to correlate the fold change obtained in the PCR array with disease aggressiveness. The cell lines – MCF7, EVSA-T, HCC-1954 and MDA-MB-231 – were designated with numbers 1 to 4, respectively. Up-regulated miRNAs are marked with a red thermometer and downregulated with a blue thermometer. Red arrows indicate an inhibition effect, and green arrows indicate an activation effect. [file Image_1.png]

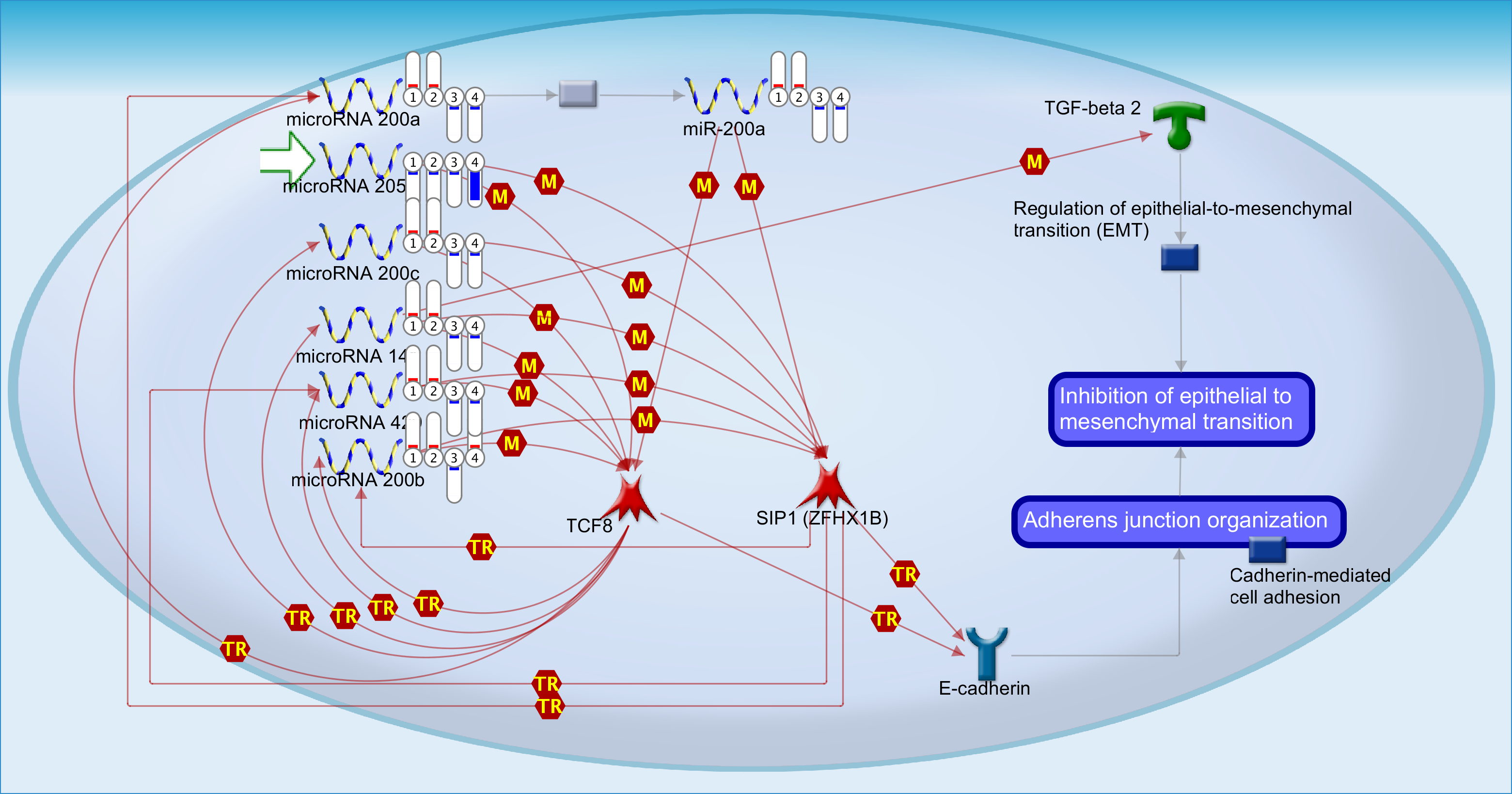

Supplement: Supplementary Figure 2 — Pathway map - MicroRNA-dependent inhibition of EMT. Pathway map generated by MetaCore™ software. The lists of DE miRNAs were uploaded into the software and compared to correlate the fold change obtained in the PCR array with disease aggressiveness. The cell lines - MCF7, EVSA-T, HCC-1954 and MDA-MB-231 - were designated with numbers 1 to 4, respectively. Up-regulated miRNAs are marked with a red thermometer and downregulated with a blue thermometer. Red arrows indicate an inhibition effect, and green arrows indicate an activation effect. [file Image_2.png]

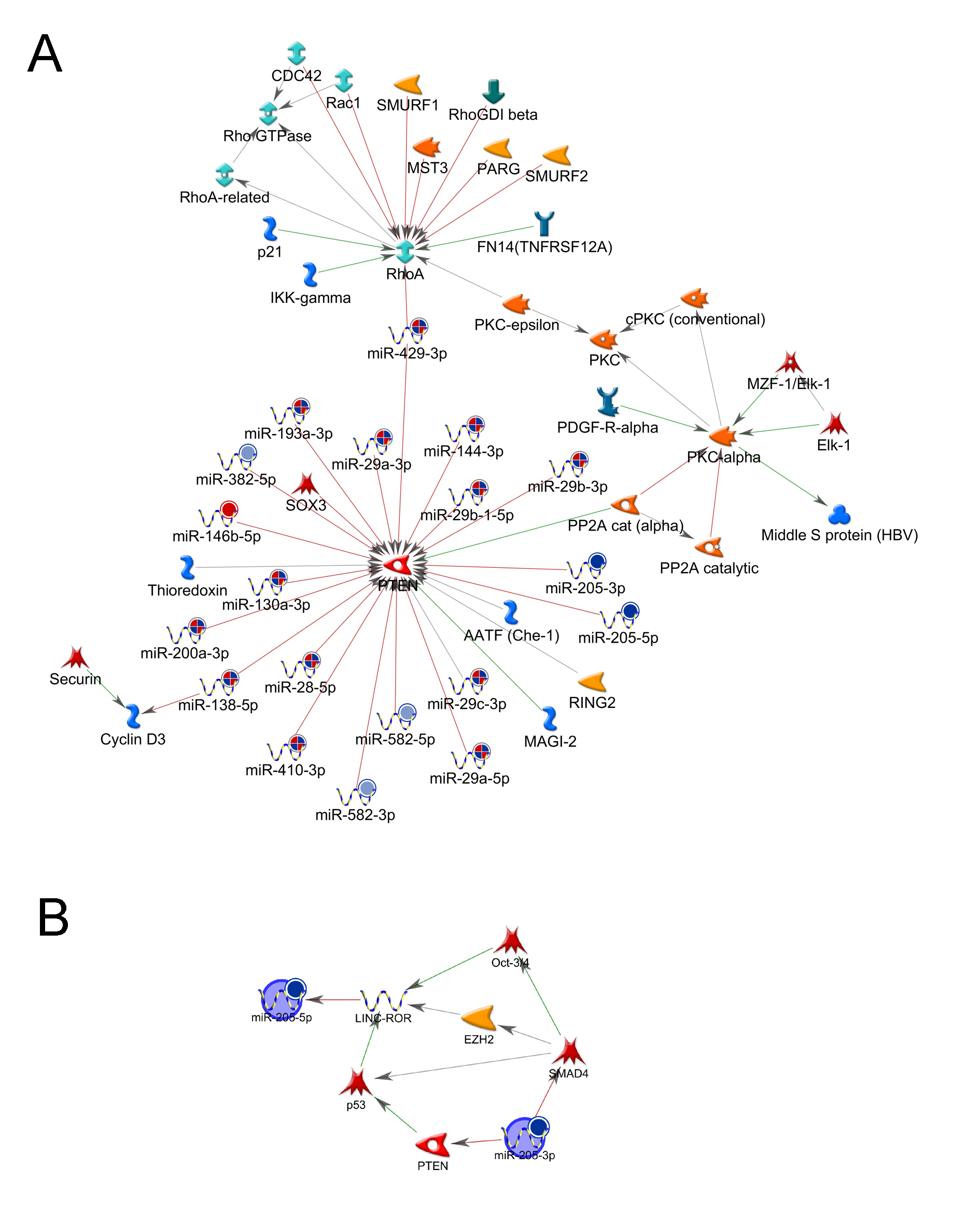

Supplement: Supplementary Figure 3 — Interaction maps in breast cancer miRNome. Representative interaction maps generated by MetaCore™ software in (A) PTEN network from DE miRNAs and (B) the interaction map of miR-205-5p. Up-egulated miRNAs are marked with red circles, and downregulated miRNAs are marked with blue circles. The ‘checkerboard’ color indicates mixed expression for the gene between files or between multiple tags for the same miRNA. [file Image_3.tiff]

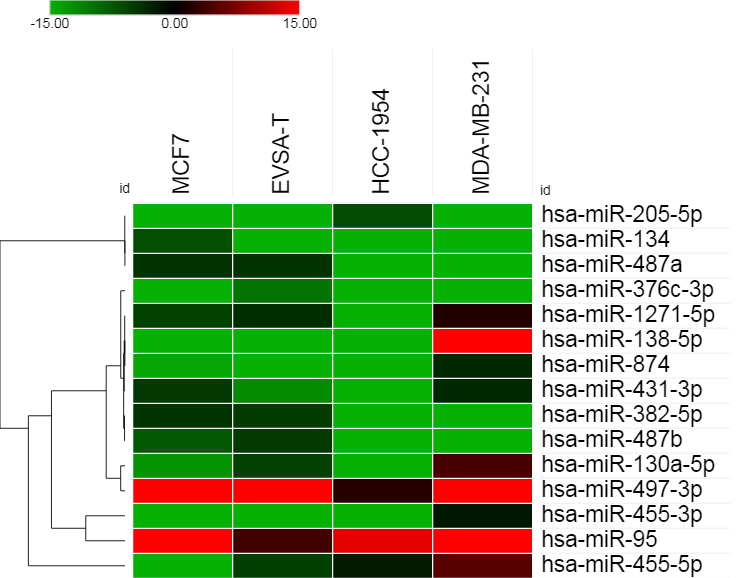

Supplement: Supplementary Figure 4 — Heatmap of 15 differentially expressed miRNAs among BC cell lines confirmed in BC subtypes (TCGA). [file Image_4.png]
